# Sodium Carboxymethyl Cellulose-Stabilised Multiple Emulsions with pH-Sensitive Behaviour, Enhanced Stability and Mucoadhesion for Oral Delivery of Chemopreventive Agents

**DOI:** 10.3390/pharmaceutics17111401

**Published:** 2025-10-29

**Authors:** Agnieszka Markowska-Radomska, Konrad Kosicki, Ewa Dluska

**Affiliations:** 1 Faculty of Chemical and Process Engineering, Warsaw University of Technology, Warynskiego 1, 00-645 Warsaw, Poland; ewa.dluska@pw.edu.pl; 2 Institute of Genetics and Biotechnology, Faculty of Biology, Warsaw University, Miecznikowa 1, 02-096 Warsaw, Poland; km.kosicki@uw.edu.pl

**Keywords:** sodium carboxymethyl cellulose, multiple emulsions, resveratrol, selenium, chemoprevention, oral delivery, mucoadhesion, pH-responsive systems

## Abstract

**Background/Objectives:** The oral administration of chemopreventive agents for colorectal cancer (CRC) remains limited by their low solubility, instability, and limited intestinal absorption. This study develops sodium carboxymethyl cellulose (CMC)-stabilised water-in-oil-in-water (W/O/W) multiple emulsions as pH-responsive carriers for co-delivery of resveratrol and selenium—two complementary chemopreventive compounds. **Methods:** Multiple emulsions differing in droplet size (small-droplet emulsions, SDE; large-droplet emulsions, LDE) and CMC concentration (0.0–0.5% *w*/*w*) are prepared in a Couette–Taylor Flow contactor. The study involves physicochemical characterisation of emulsions (droplet size, stability, rheological behaviour, ζ-potential, encapsulation efficiency), evaluation of release profiles under simulated gastric pH (2.0) and intestinal pH (7.0) conditions, including pathological environments (pH = 5.5), and ex vivo assessment of mucoadhesion using porcine intestinal tissue. **Results:** SDE and LDE containing CMC (0.0–0.5% *w*/*w*) exhibit a complex “drop-in-drop” structure, with Sauter mean diameters of approximately 9–12 μm and 23–25 μm, respectively, and high encapsulation efficiencies (>91%). Increasing CMC concentration enhances viscosity and induces more negative ζ-potential, confirming polymer adsorption at the oil–water interface. Under simulated gastric pH = 2.0, compound release remains limited (≤15%), whereas gradual/sustained release is observed under simulated intestinal pH (5.5/7.0). Mucoadhesion increases with polymer concentration, reaching ~90% for SDE and ~70% for LDE at 0.5% *w*/*w* CMC, and remains above 50% under simulated pathological conditions. **Conclusions:** The study demonstrates that CMC incorporation improves the structural stability, modulates the release behaviour, and enhances the mucoadhesive properties of W/O/W multiple emulsions. These findings suggest that CMC-stabilised emulsions may be further explored as oral delivery vehicles for CRC chemoprevention.

## 1. Introduction

The effective oral delivery of chemopreventive agents remains a key challenge in pharmaceutical and nutraceutical formulation development. Chemoprevention has gained attention as a complementary approach for colorectal cancer (CRC) prevention [1,2]. Various agents, including anti-inflammatory drugs, metabolic regulators, antioxidants, and micronutrients, have been explored for their chemopreventive potential, either individually or in combination, acting synergistically through complementary molecular pathways involved in inflammation, oxidative stress, and tumourigenesis [2,3,4].

Many bioactive compounds with chemopreventive potential, including polyphenols and micronutrients, exhibit physicochemical or biological limitations that reduce oral bioavailability [1,3,4]. Among these, resveratrol demonstrates poor aqueous solubility, chemical instability, and susceptibility to degradation in the gastrointestinal tract, whereas selenium shows variable absorption and bioavailability. Therefore, improving chemopreventive agents’ stability and intestinal uptake is essential to overcome these limitations in oral delivery system design.

Various formulation strategies have been developed to overcome these limitations [5,6,7,8]. Lipid-based carriers such as nanoemulsions, solid lipid nanoparticles (SLNs), and nanostructured lipid carriers (NLCs), as well as polymer-based systems including enteric-coated microspheres, hydrogels, and polymer–lipid hybrid particles, have demonstrated improved solubility and protection [5,6]. However, these systems often suffer from limited encapsulation efficiency for compounds of dual solubility, uncontrolled release, or structural instability under gastrointestinal conditions. More recent approaches employ multicomponent carriers combining physical protection with responsiveness to environmental stimuli (pH or enzymatic activity) to achieve targeted and sustained release [7,8]. Within this context, water-in-oil-in-water (W/O/W) multiple emulsions have emerged as promising multi-compartment delivery systems for oral administration. Their hierarchical structure enables the simultaneous encapsulation of hydrophilic and lipophilic compounds in distinct internal and membrane phases, ensuring protection and controlled release [9,10,11]. Multiple emulsions have been widely investigated for food, cosmetic, and pharmaceutical applications [10,12,13]. Hydrocolloids are frequently introduced into the external aqueous phase to enhance the stability of emulsions. Among them, carboxymethyl cellulose and its derivatives, e.g., sodium carboxymethyl cellulose (CMC), are particularly effective due to their reversible, non-covalent stabilisation mechanism, which enables interfacial control and adaptation to varying pH environments [13,14,15]. As a food-grade anionic polysaccharide, CMC increases emulsion viscosity, forms cohesive interfacial films, and provides steric and electrostatic stabilisation [16,17,18]. The pH-dependent ionisation of CMC allows modulation of surface charge and interfacial organisation in response to environmental changes [8,16]. These properties make CMC a suitable stabiliser for responsive oral delivery systems that are capable of protecting bioactives during gastric transit and facilitating targeted intestinal release. Although CMC is widely used for multiple emulsion stabilisation, the mechanistic relationship between interfacial behaviour and functional performance, particularly regarding stability, release, and mucoadhesion, remains insufficiently elucidated.

**Figure 1:**
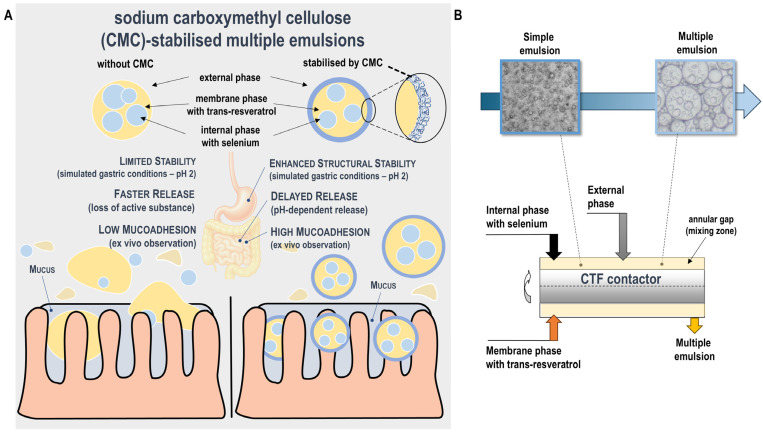
(**A**) A comparative schematic shows the role of sodium carboxymethyl cellulose (CMC) in stabilising W/O/W multiple emulsions. CMC forms an interfacial polymer layer that improves structural stability, modulates pH-responsive release, and enhances mucoadhesion to intestinal mucus, indicating potential advantages for oral delivery and preservation of formulation integrity within the intestinal tract. (**B**) Schematic representation of the one-step preparation of water-in-oil-in-water (W/O/W) multiple emulsions in a Couette–Taylor flow (CTF) contactor.

This study investigates the physicochemical and functional behaviour of CMC-stabilised (W/O/W) multiple emulsions as oral carriers for resveratrol and selenium (compounds with recognised chemopreventive roles in CRC) (Figure 1A). CMC concentration in the external aqueous phase was systematically varied to evaluate its influence on interfacial polymer adsorption, ζ-potential, and droplet size during sequential exposure to simulated gastric (pH 2) and intestinal (pH 7) conditions. The release characteristics of co-encapsulated compounds and the mucoadhesive performance of emulsions were further examined under physiological and pathological simulated gastrointestinal environments. The experiments were conducted under simplified gastrointestinal conditions using enzyme-free and bile salt-free media and an ex vivo mucoadhesion model. These conditions allowed controlled analysis of interfacial phenomena and polymer behaviour without interference from biological variability. Although they do not fully reproduce the complexity of the gastrointestinal environment, they enable reproducible characterisation of key formulation parameters. The findings provide mechanistic insight into how CMC contributes to interfacial stabilisation, pH responsiveness, controlled release, and mucoadhesion, offering a basis for further in vitro studies under biorelevant conditions and in vivo validation of CMC-stabilised multiple emulsions for oral chemopreventive delivery.

## 2. Materials and Methods

### 2.1. Materials

Emulsifying and stabilising agents included Pluronic P123, Span 83, and sodium carboxymethyl cellulose (CMC, high viscosity) (Sigma-Aldrich GmbH, Steinheim, Germany). Powdered gelatin, pure p.a., was obtained from Avantor (Gliwice, Poland), and Tween 80 was purchased from Fisher Scientific Company LLC. (Fair Lawn, NJ, USA). Active compounds (trans-resveratrol and sodium selenite) were supplied by Sigma-Aldrich (Warsaw, Poland). Refined sesame oil was sourced from Caesar & Loretz GmbH (Hilden, Germany).

Chemicals, including sodium bicarbonate, sodium chloride, sodium hydroxide (0.1 M), hydrochloric acid (0.1 M), potassium chloride, sodium deoxycholate, calcium chloride dihydrate, monosodium phosphate, ammonium carbonate, magnesium chloride hexahydrate, N-acetylcysteine (NAC), hydrochloric acid (1 M), phenol (80%), ethanol (50%), and sulphuric acid (96%), were purchased from Avantor (Gliwice, Poland). Phosphate-buffered saline (PBS) and 2-mercaptobenzothiazole (MTB) were supplied by Sigma-Aldrich (Warsaw, Poland). High-purity Milli-Q water was used throughout. All solvents were of analytical grade.

### 2.2. Preparation of Multiple Emulsions

Water-in-oil-in-water (W/O/W) multiple emulsions were prepared using a single-step emulsification method in a Couette–Taylor flow (CTF) contactor operating in the horizontal position [19,20]. The apparatus consisted of two concentric cylinders forming an annular gap (inner cylinder diameter: 0.035 m, gap width: 1.5 mm, length: 0.4 m), with the inner cylinder rotating and the outer one stationary. Emulsification and encapsulation of bioactive compounds co-occurred. The internal and membrane phases were introduced through the inlet section of the contactor, while the external aqueous phase was supplied further downstream. The process is illustrated schematically in Figure 1B.

Two series of multiple emulsions were formulated: small-droplet emulsions (SDE) and large-droplet emulsions (LDE). Within each series, the formulations differed in sodium carboxymethyl cellulose (CMC) concentration in the external aqueous phase (0.0, 0.1, 0.3, and 0.5% *w*/*w*). Process parameters were adjusted to maintain comparable droplet sizes and distributions of the membrane phase across formulations, irrespective of CMC concentration. All emulsions were prepared in triplicate. Samples were stored in dark glass bottles at 25 °C for 90 days to assess structural stability. Detailed information on the phase composition and emulsification parameters is presented in Table 1 and Table 2.

Trans-resveratrol was incorporated into the internal phase of the emulsion (1.5 mg per 100 cm^3^ emulsion; 65.7 µM), while selenium (sodium selenite) was introduced into the membrane phase (55 µg per 100 cm^3^ emulsion; 3.18 µM) as chemopreventive agents. The selected concentrations ensured emulsion stability and corresponded to sub-cytotoxic, biologically active levels previously reported for intestinal epithelial and colorectal cell models. Resveratrol at approximately 40 µM has been shown to inhibit NF-κB-mediated inflammatory responses in colon cells [21], while sodium selenite within the 1–10 µM range modulates redox homeostasis and induces protective cellular signalling without cytotoxicity [22,23]. These loading levels were therefore intended to reproduce local intestinal exposure conditions relevant to CRC chemoprevention.

The obtained SDE and LDE were subsequently subjected to physicochemical characterisation and evaluation under simulated gastrointestinal transit conditions.

### 2.3. Microscopic Observation and Droplet Size Analysis

The morphology of the multiple emulsions was examined using an Olympus BX60 optical microscope equipped with an Olympus SC50 digital camera (Olympus, Tokyo, Japan). A thin layer of the emulsion was placed directly on a microscope slide without a cover glass. The emulsion structure was assessed based on representative images selected from a large dataset [19,20].

Droplet size analysis was conducted using Image-Pro Plus 4.5 software (Media Cybernetics, Rockville, MD, USA). Droplet diameters were measured using the three-point method along the droplet circumference. The mean Sauter diameter was determined for the membrane phase droplets (*D*_32_) and the internal aqueous phase droplets (*d*_32_).

Microscopic observation was also employed to assess the stability of the emulsions stored in amber glass bottles at 25 °C. Structural changes and droplet size evolution were monitored during storage and re-evaluated after 90 days.

### 2.4. Encapsulation Efficiency Measurements

The encapsulation efficiencies of the chemopreventive agents, trans-resveratrol (*EE_r_*) and selenium (*EE_S_*), were determined based on the concentration of free, non-encapsulated compounds presented in the continuous phase of the emulsions immediately after preparation. The analysis was conducted according to the procedure described in Section 2.7. The dispersed and continuous phases were separated by filtration through a regenerated cellulose syringe filter (0.45 µm; Profill, Třinec, Czech Republic). The encapsulation efficiency (*EE*) was calculated as the percentage of the total active ingredient successfully entrapped within the emulsion droplets, according to Equation (1).

(1) $$EE=\left(M_{0}-M_{t}\right)\cdot {\left(M_{0}\right)}^{-1}\cdot 100\%$$

where *M_t_* is the mass of non-encapsulated compound in the external phase of emulsion; *M*_0_ is the initial mass of the compound added during emulsion preparation.

### 2.5. Rheological Measurements

The rheological behaviour of the emulsions was examined using a RheolabQC rotational viscometer (Anton Paar, Graz, Austria) with a DG42 double-gap concentric cylinder system. Flow curves were recorded at shear rates ranging from 50 to 1500 s^−1^ at 37 °C, corresponding to the physiological temperature.

### 2.6. ζ-Potential Measurements

The ζ-potential of CMC-stabilised multiple emulsions was determined using a Zetasizer Nano S series (Malvern Instruments, Malvern, UK) equipped with a DTS1070 capillary cell. Emulsion samples were diluted to 0.005% *w*/*w* with phosphate-buffered saline (PBS) at the corresponding pH values (pH 7.4, 7.0, and 5.5 mimicking initial, physiological, and pathological intestinal conditions, respectively) to minimise multiple scattering effects. Measurements were performed at 25 °C. Each sample was equilibrated for 2 min prior to analysis.

### 2.7. Chemopreventive Agents Quantification

Substance-specific methods were employed to determine the concentrations of chemopreventive agents in the external emulsion phase (encapsulation efficiency determination) and in simplified simulated gastric and intestinal fluids (release studies).

Resveratrol was quantified according to [24]. Absorbance was measured at 305 nm, and concentrations were calculated from calibration curves (Figure S1, Table S1—Supplementary Materials) prepared from freshly made standard solutions in the same solvent system.

Selenium (originating from sodium selenite) was analysed following the procedure of Bera and Chakrabartty [25] with modifications. The pH of the samples was adjusted to 7.5 ± 0.2 with 0.1 M HCl/NaOH to optimise the formation of a yellow selenium complex with 2-mercaptobenzothiazole. For analysis, 1 cm^3^ of the sample or standard solution was mixed with 15 cm^3^ of 12 M HCl and 4 cm^3^ of a 0.1% (*w*/*v*) solution of 2-mercaptobenzothiazole (MTB) in 50% ethanol. The mixture was diluted with 2 M HCl, incubated for 10 min at room temperature, and the absorbance was measured at 370 nm against a reagent blank. Selenium concentrations were obtained from calibration curves. The method was selected for simple, aqueous, enzyme- and bile salt-free media representative of the model systems used, where matrix interferences are minimal. The modified assay was validated and confirmed suitable for quantitative selenium determination in the tested matrices (see Supplementary Materials, Section S2: Figures S2 and S3, Tables S2 and S3). The continued analytical relevance of spectrophotometric complex-forming methods for selenium determination has been confirmed in more recent reviews [26].

All spectrophotometric measurements were carried out using a Jasco FP-6500 spectrophotometer (Tokyo, Japan).

### 2.8. Simulated Gastrointestinal Transit (Git) Conditions of Emulsion

The emulsions were subjected to simulated gastrointestinal conditions to replicate the sequential pH transition during oral transit. The experiments were performed in enzyme-free media to eliminate potential interferences and ensure reliable analytical measurements. Simplified simulated gastric and intestinal fluids were prepared by adjusting phosphate-buffered saline (PBS) to the desired pH without the addition of pepsin, pancreatin, or bile salts. The pH of the incubation medium was initially adjusted to 2.0 (gastric phase) using 0.1 M HCl and subsequently increased to 7.0 (intestinal phase) after 2 h with 0.1 M NaOH, reflecting the pH shift encountered during oral digestion. The emulsions, diluted as required for each analysis, were incubated at 37 °C under gentle agitation (100 rpm, mimicking gastrointestinal motility) for 4 h (2 × 2 h), corresponding to typical gastric and intestinal residence times. This approach reproduces gastrointestinal fluids’ physiological pH and ionic strength while avoiding matrix effects associated with enzymatic or protein components.

### 2.9. Emulsion Morphology Under Simulated Gastrointestinal Transit Conditions

The morphology of the emulsions was evaluated under simulated GIT conditions by analysing changes in droplet size of the membrane phase after exposure to simplified gastric (pH 2.0) and intestinal (pH 7.0) environments (dilution ratio: 1:10 *v*/*v*, emulsion–medium). The measurements were performed according to the procedure described in Section 2.3.

### 2.10. Determination of Interfacial CMC Concentration Under Simulated GIT Conditions

The interfacial concentration of CMC (Γ) was determined to assess polymer adsorption at the droplet surface under the simulated GIT conditions described above. The sequential pH transition followed the procedure outlined in Section 2.7, with emulsions diluted 1:1 (*v*/*v*) with phosphate-buffered saline (PBS). At each stage (after 2 h at pH 2 and a further 2 h at pH 7), samples were withdrawn and centrifuged (5000 rpm, 15 min) to separate the external aqueous phase containing unadsorbed polymer. The concentration of unadsorbed CMC in the supernatant was quantified using a modified phenol–sulphuric acid colourimetric method [27,28]. In brief, 2 cm^3^ of the supernatant was mixed with 0.05 cm^3^ of 80% phenol and 5 cm^3^ of concentrated sulphuric acid (98%), vortexed, and allowed to react for 30 min at room temperature. The absorbance of the resulting orange-coloured solution was recorded at 490 nm using a spectrophotometer (Jasco FP-6500, Tokyo, Japan).

The adsorbed CMC at the droplet interface was calculated as the difference between the initially introduced CMC concentration (*C_in_*) and the residual CMC in the supernatant (*C_s_*). The interfacial concentration (*Γ*, mg·m^−2^) was obtained according to Equation (2):

(2) $$\Gamma =\left(C_{in}-C_{s}\right)\cdot D_{32}{\cdot \left(6\Phi \right)}^{-1}$$

where *D*_32_ is the Sauter mean diameter of the membrane-phase droplets, and *Φ* is the dispersed-phase volume fraction.

### 2.11. In Vitro Release Study of Chemopreventive Agents in Simulated Gastrointestinal Transit

The release behaviour of chemopreventive agents from the emulsions was evaluated under simulated GIT pH conditions. Emulsion samples (5 cm^3^) were diluted with the simulated medium at a 1:10 (*v*/*v*; emulsion–medium) ratio. At predetermined time points during each stage (gastric (pH 2.0) and intestinal (pH 7.0)), aliquots were collected and filtered through a 0.45 μm regenerated cellulose syringe filter (Profill, Czech Republic). The study was also extended to simplified pathological GIT conditions, consisting of an initial gastric phase at pH 2.0, followed by the intestinal phase adjusted to pH 5.5. The filtrates were analysed to determine the concentrations of released resveratrol and selenium according to the analytical procedures described in Section 2.7.

The release profiles were expressed as the cumulative percentage of compound released over time relative to the initial loading.

### 2.12. Ex Vivo Investigation of Intestinal Mucoadhesion Under Physiological and Pathological Conditions

The mucoadhesive behaviour of the emulsions was evaluated using a modified falling liquid film technique described by Komati et al. (2019), Winter et al. (2020) [29,30]. Freshly excised porcine small intestines were obtained from a local slaughterhouse within one hour of slaughter. The material was obtained as a by-product of the routine slaughter of animals intended for food production. The tissues were cut into 5 cm segments, slit longitudinally, rinsed with sterile 0.9% sodium chloride solution for 30 min, and mounted on a semicylindrical support inclined at 45° to expose the mucosal surface.

For assays under physiological intestinal conditions, 0.2 cm^3^ of emulsion was applied uniformly to the mucosal surface and allowed to interact for 15 min at 37 °C. The mounted tissue was then washed with phosphate-buffered saline (PBS, pH 7) using a peristaltic pump Masterflex L/S (2 cm^3^·min^−1^, 20 min). The washing fluid containing the non-adhered emulsion was collected, and the concentration of the emulsion in the effluent was determined from the concentration–refractive index calibration curves [31]. The percentage of mucoadhesion was calculated according to Equation (3):

(3) $$\%\;\mathrm{of}\;\mathrm{mucoadhesion}=\left(m_{\mathrm{initial}}-m_{\mathrm{NA}}\right)\cdot {\left(m_{\mathrm{initial}}\right)}^{-1}\cdot 100\%$$

(*m_initial_*—total mass of emulsion applied at the start, *m_NA_*—mass of the non-adhered emulsion recovered in the washings.)

Microscopic observations of the intestinal samples were also performed using an Olympus BX50 optical microscope.

Simulation of pathological intestinal conditions: In addition to assays under physiological intestinal conditions, three pathological variants were examined to simulate factors that may affect mucoadhesion during intestinal inflammation or dysfunction. Freshly excised porcine small intestine was used as the substrate in all cases, with the following modifications:

(1) Acidic pH environment (pH 5.5): To reproduce the mildly acidic microenvironment associated with intestinal inflammation, which alters mucin conformation and reduces CMC ionisation, the intestinal segments were preincubated for 15 min in PBS adjusted to pH 5.5 using 0.1 M HCl [32]. During washing, PBS (pH 5.5) was used instead of neutral buffer to maintain consistent conditions throughout the assay.
(2) Reduced mucus layer: To mimic the thinning of the mucus layer typical of inflammatory bowel disease, the mucosal surface was rinsed three times with PBS (pH 7.0) containing 0.5% *w*/*v* N-acetylcysteine (NAC), followed by a 10 min incubation at 37 °C to loosen and partially remove adherent mucins [33]. The tissue was washed thrice with PBS to remove residual NAC before applying the emulsion.
(3) Increased bile salt concentration: Pathological bile reflux and malabsorption were simulated by supplementing the washing buffer with sodium deoxycholate (10 mM) during the 20 min peristaltic washing step. This concentration corresponds to the upper physiological range observed under pathological bile salt exposure [34].

All other experimental parameters were identical to those applied under physiological conditions.

Each experimental condition (physiological and pathological variants) was evaluated using four independent biological replicates (*n* = 4), each obtained from a different intestinal segment of a different animal. All measurements were performed in technical triplicate to ensure analytical reproducibility.

### 2.13. Statistical Analysis

All data are expressed as the mean ± standard deviation (±SD). All experiments were performed and analysed in triplicate (unless otherwise stated). Statistical analysis was performed using GraphPad Prism (version 10.0, GraphPad Software, San Diego, CA, USA). The normality of data distribution was verified using the Shapiro–Wilk test. Differences between groups were analysed by one-way analysis of variance (ANOVA) followed by Tukey’s post hoc test for multiple comparisons. A *p*-value < 0.05 was considered statistically significant.

## 3. Results and Discussion

### 3.1. Emulsion Characterisation

**Figure 2:**
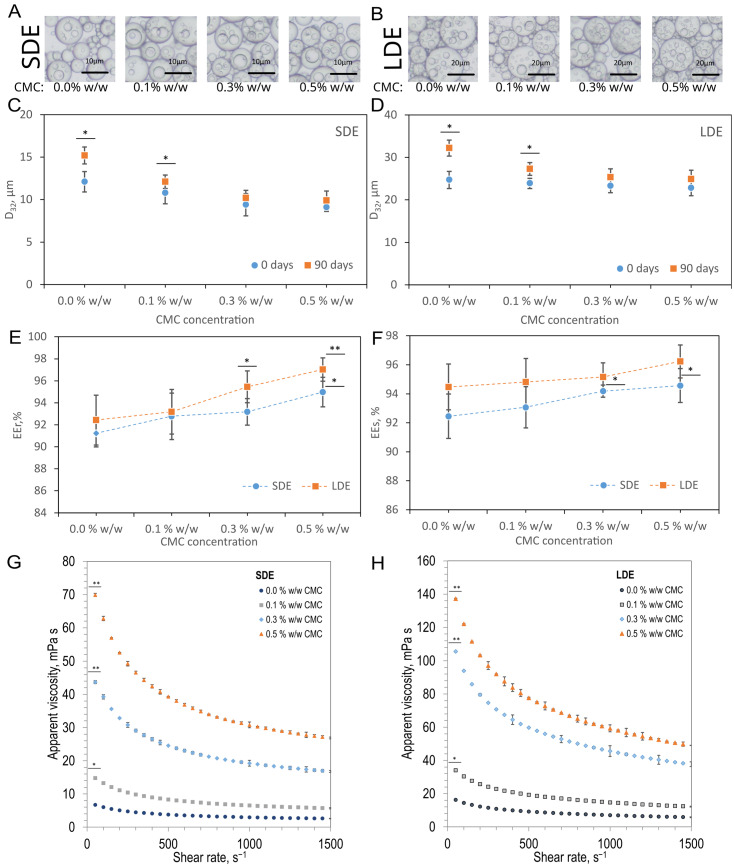
Effect of sodium carboxymethyl cellulose (CMC) concentration on the physicochemical characteristics of small-droplet (SDE) and large-droplet (LDE) W/O/W multiple emulsions containing various CMC concentrations: representative optical micrographs of SDE (**A**) and LDE (**B**) emulsions; the mean droplet diameter (*D*_32_) of the membrane phase droplets measured immediately after preparation (0 days) and after 90 days of storage for SDE (**C**) and LDE (**D**) emulsions (asterisks indicate significant differences relative to initial values for each formulation; *p* < 0.05; one-way ANOVA with Tukey’s test); encapsulation efficiencies of trans-resveratrol (*EEᵣ*) (**E**) and selenium (*EEₛ*) (**F**) (asterisks indicate significant differences compared with CMC-free samples; *p* < 0.05 (*), *p* < 0.01 (**); one-way ANOVA with Tukey’s test); apparent viscosity of SDE (**G**) and LDE (**H**) emulsions as a function of shear rate (significant differences at 50 s^−1^ vs. CMC-free samples; *p* < 0.05 (*), *p* < 0.01 (**); two-way ANOVA with Tukey’s test). Each value represents the mean ± SD (error bars not visible, mean errors are the same size, or smaller, than the symbol for a given measurement).

Two groups of W/O/W multiple emulsions were obtained: small-droplet emulsions (SDE) and large-droplet emulsions (LDE) (Figure 2A,B). The concentration of CMC in the external phase was varied (0–0.5% *w*/*w*) for both LDE and SDE, and the rotational frequency of the rotor in the Couette–Taylor flow apparatus was adjusted to maintain comparable droplet sizes within each group, while preserving the overall size difference between LDE and SDE (Table 2).

The mean diameters (*D*_32_) of the membrane phase droplets were approximately 9–12 µm for SDE and 23–25 µm for LDE (Figure 2C,D). The variations in *D*_32_ within each group (SDE, LDE) did not exceed 2–3 μm. The LDE were characterised by a higher dispersed phase volume fraction (0.6) compared with SDE (0.5). The internal phase droplet size (*d*_32_) remained consistent across all formulations, averaging 3–5 μm. These parameters were carefully controlled to ensure that subsequent differences observed under simulated gastrointestinal conditions (pH changes) or in mucoadhesion assays (ex vivo study) could be attributed primarily to the presence and concentration of CMC. After 90 days of storage, emulsions without CMC exhibited the most significant increase in droplet size (Δ*D*_32_ ~3–7.5 μm), and formulations containing 0.1% *w*/*w* CMC also showed significant but smaller droplet growth (*p* < 0.05). In contrast, formulations containing ≥ 0.3% *w*/*w* CMC showed only minor, statistically insignificant changes (Δ*D*_32_ < 3 μm), indicating that CMC effectively limited droplet growth and coalescence.

The encapsulation efficiencies (*EE*) for trans-resveratrol (*EEᵣ*) and selenium (*EEₛ*) were above 91% across all formulations (Figure 2E,F). Both emulsion type and CMC concentration significantly affected encapsulation efficiency (*EE*) values (*p* < 0.01). Higher CMC (≥0.3% *w*/*w*) concentrations produced modest but statistically significant improvements in both EEᵣ and EEₛ (*p* < 0.05). Specifically, SDE with 0.5% *w*/*w* CMC reached 95.0 ± 1.3% (*EEᵣ*) and 95.9 ± 1.2% (*EEₛ*), while LDE with 0.5% CMC achieved 97.0 ± 1.1% (*EEᵣ*) and 96.2 ± 1.1% (*EEₛ*). The results confirm that the emulsification process ensured efficient entrapment of both active compounds, while CMC addition provided an additional stabilising effect.

Apparent viscosity measurements (Figure 2G,H) revealed typical shear-thinning behaviour for all emulsions. At 50 s^−1^, viscosities ranged from 6.7 mPa·s (SDE, CMC-free) to 70.0 mPa·s (SDE, 0.5% *w*/*w* CMC), and from 16.2 mPa·s (LDE, CMC-free) to 137.0 mPa·s (LDE, 0.5% *w*/*w* CMC). Both emulsion type and CMC concentration significantly affected viscosity at 50 s^−1^ (*p* < 0.001), with significant increases observed from 0.1% CMC in LDE and 0.3% CMC in SDE. The larger droplet size and higher dispersed-phase fraction of LDE resulted in consistently higher viscosities at all shear rates. The effect of CMC was evident in both groups, but the relative increase was more pronounced in LDE, reflecting stronger hydrodynamic interactions between the larger oil droplets and the CMC-enriched continuous phase.

Measured viscosity values were comparable to those reported for physiological fluids within the gastrointestinal tract. Human saliva typically exhibits shear-rate-dependent viscosities ranging from approximately 1 to 10 mPa·s. In comparison, intestinal mucus can reach values from several tens to several hundred mPa·s depending on hydration, composition, and pH [35,36,37,38]. Emulsions containing higher CMC concentrations reached viscosities overlapping with the lower boundary of those observed for mucus and gastric chyme. This polymer-induced viscosity enhancement likely increases resistance to shear forces, contributing to microstructural stability by reducing droplet mobility and collision frequency. Such rheological behaviour may also favour longer residence and improved mucoadhesive potential within the intestinal environment.

When compared with the International Dysphagia Diet Standardisation Initiative (IDDSI), which defines viscosity thresholds at 50 s^−1^ for tailoring liquid textures in individuals with swallowing disorders (e.g., elderly individuals, children, and those with neurological or oncological conditions) [39], most of the obtained emulsions can be classified as “thin” (<50 mPa·s). The incorporation of higher CMC concentrations (≥ 0.3% *w*/*w*) increased viscosity to levels approaching the “nectar-like” range (51–350 mPa·s) (Figure 2G,H), which is considered easier to control during swallowing. These findings indicate that CMC addition not only enhances the rheological stability of the emulsions under gastrointestinal shear but also enables adjustment of the emulsions’ viscosity towards safer, more physiologically relevant consistencies for the dysphagic population.

### 3.2. pH-Dependent Behaviour of Emulsions Under Simplified Simulated Gastrointestinal Conditions

#### 3.2.1. Droplet Size Evolution

**Figure 3:**
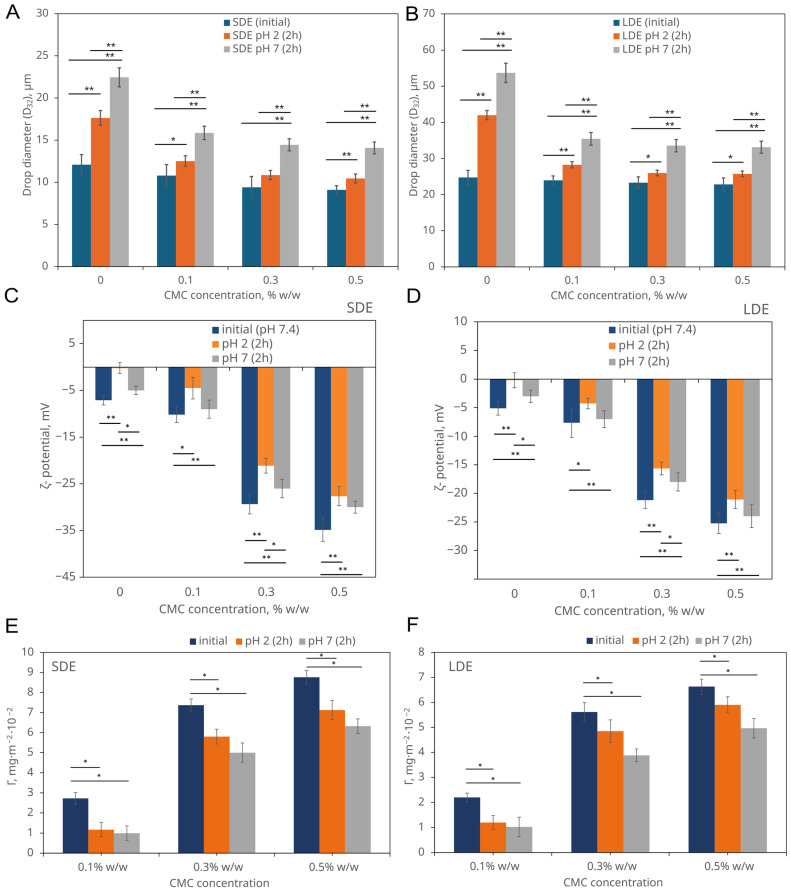
Changes in the physicochemical properties of CMC-stabilised multiple emulsions during simulated gastrointestinal pH transition (pH 2.0 → 7.0). (**A**,**B**) Sauter mean diameter (*D*_32_) of the membrane-phase droplets in small-droplet (SDE) and large-droplet (LDE) emulsions immediately after preparation and after sequential exposure to gastric (pH 2.0) and intestinal (pH 7.0) media. (**C**,**D**) *ζ*-potential of SDE and LDE under identical pH-transition conditions, showing reduced surface charge at pH 2.0 (due to protonation of CMC carboxyl groups) and partial recovery at pH 7.0. (**E**,**F**) Interfacial CMC concentration (Γ) of SDE and LDE following sequential pH 2.0 and pH 7.0 exposure. Asterisks indicate statistically significant differences between pH stages or relative to initial values (*p* < 0.05 (*), *p* < 0.01 (**); one-way ANOVA with Tukey’s post hoc test). (*p* < 0.05 (*), *p* < 0.01 (**); one-way ANOVA with Tukey’s test). Each value represents the mean ± SD (error bars not visible, mean errors are the same size, or smaller, than the symbol for a given measurement).

Figure 3A,B show the changes in the Sauter mean diameter (*D*_32_) of the membrane phase droplets at pH 2.0 and 7.0, illustrating the effect of sequential exposure to pH variation under simulated gastrointestinal conditions.

After 2 h under pH 2-gastric conditions, droplet size increased moderately (by approximately 20–40% compared with the initial value), reaching 10.5–17.6 µm. Subsequent transfer to intestinal conditions (pH 7.0) caused further enlargement, with *D*_32_ ranging from 14.0 to 22.5 µm. The most pronounced growth was observed in CMC-free samples, where *D*_32_ increased from 12.1 to 22.5 µm. In contrast, emulsions containing 0.3–0.5% *w*/*w* CMC exhibited a smaller, though statistically significant, increase, indicating improved interfacial resistance to coalescence and flocculation. A similar trend was observed for LDE, which showed higher initial *D*_32_ values. After 2 h at pH 2.0, mean diameters increased to 25.7–42.0 µm and further to 33.1–53.7 µm following 2 h at pH 7.0. These results confirm that CMC enhances the structural stability of multiple emulsions against pH-induced coalescence in a concentration-dependent manner.

The smaller SDE droplets exhibited significantly lower relative increases in *D*_32_ compared with LDE in CMC-free formulations (*p* < 0.05; two-way ANOVA with Tukey’s test, Figure S4), reflecting their greater resistance to pH-induced destabilisation. This difference between emulsion types was largely eliminated at higher CMC concentrations, indicating that CMC effectively stabilised both systems.

The addition of CMC markedly reduced droplet growth in both emulsion types, with the strongest effects observed at 0.3–0.5% *w*/*w*. CMC acts as both a steric and rheological stabiliser of the continuous phase, reducing droplet motion and limiting collision frequency [15,17]. Similar protective effects have been reported for other food-grade emulsions, where CMC increased viscosity, restricted droplet mobility, and delayed destabilisation during simulated gastric digestion [7,18]. The observed droplet-size evolution reflects the combined action of CMC adsorption, viscosity enhancement, and electrosteric stabilisation, which together maintain the integrity of the multiple-emulsion structure under varying gastrointestinal pH conditions.

#### 3.2.2. ζ-Potential Analysis of Emulsions

Figure 3C,D show the ζ-potential values of SDE and LDE measured under initial (pH 7.4), gastric (pH 2.0), and intestinal (pH 7.0) conditions. The surface charge of the emulsions depended strongly on both CMC concentration and droplet size. In all formulations, ζ-potential became increasingly negative with higher CMC levels, indicating progressive adsorption of the anionic polymer onto the droplet surface.

For SDE, initial ζ-potentials ranged from approximately −10 mV at 0.1% *w*/*w* CMC to −35 mV at 0.5% *w*/*w* CMC. Exposure to gastric pH conditions (pH 2.0) markedly reduced the surface charge (−4 to −25 mV), reflecting protonation of the CMC carboxyl groups and diminished electrostatic repulsion between droplets. Upon transition to intestinal conditions (pH 7.0), ζ-potentials became slightly more negative (−7 to −30 mV), suggesting partial re-ionisation of CMC but incomplete restoration of the initial charge.

LDE followed the same general trend but exhibited less negative ζ-potentials at all corresponding concentrations (−8 to −25 mV initially, −4 to −21 mV at pH 2.0, and −6 to −24 mV at pH 7.0). This difference arises from their lower specific surface area, which limits the density of CMC adsorption at the oil–water interface.

Statistical analysis (one-way ANOVA with Tukey’s test) confirmed significant differences between the pH stages (*p* < 0.05 or *p* < 0.01), particularly between initial and intestinal conditions.

The overall relationship |ζ|(initial) > |ζ|(pH 7.0) > |ζ|(pH 2.0) was consistent across all samples, in agreement with the protonation–deprotonation behaviour of CMC reported previously [8,40,41]. The reduction in surface charge under acidic conditions is commonly attributed to protonation of carboxyl groups, whereas the partial charge recovery upon neutralisation results from re-ionisation and rearrangement of the adsorbed polymer layer.

These pH-dependent variations in ζ-potential are characteristic of polysaccharide-stabilised emulsions [9] and correspond with the observed decrease in colloidal stability under gastric conditions. The lower magnitude of ζ-potential at low CMC concentrations reduces electrostatic repulsion and facilitates partial droplet coalescence, in line with the adsorption behaviour described in Section 3.2.3. Nevertheless, despite the reduced electrostatic repulsion at acidic pH, no measurable increase in droplet diameter was observed in CMC-stabilised emulsions (Figure 3A,B). This can be attributed to the electrosteric stabilisation imparted by the adsorbed CMC layer, which remains hydrated and cohesive even upon partial protonation of its carboxyl groups (pKₐ ≈ 4–4.5). The dense polymer network effectively preserves droplet separation and interfacial integrity, preventing size variation despite changes in ζ-potential.

The relative change in ζ-potential (%|Δζ|) was significantly higher for CMC-free emulsions (*p* < 0.05; Supplementary Materials, Figure S5), further supporting the stronger surface-charge stabilisation provided by CMC and the enhanced interfacial protection of SDE compared with LDE systems.

#### 3.2.3. The Behaviour of CMC Adsorbed onto Emulsion Droplets

Figure 3E,F present the interfacial CMC concentration (*Γ*) measured in emulsions sequentially exposed to simulated gastric (pH 2) and intestinal (pH 7.0) conditions. Although CMC does not form covalent bonds with the non-polar sesame oil droplets, its adsorption at the interface is driven by physical interactions such as weak hydrophobic association, steric entanglement, and increased viscosity in the continuous phase [7].

The data show a consistent decrease in *Γ* compared with the initial values, with the magnitude of reduction depending on both CMC concentration and emulsion type. For SDE, *Γ* values decreased notably after exposure to gastric conditions (pH 2.0), from 2.72 to 1.17 mg·m^−2^ × 10^−2^ at 0.1% *w*/*w*, from 7.38 to 5.80 mg·m^−2^ × 10^−2^ at 0.3% *w*/*w*, and from 8.76 to 7.13 mg·m^−2^ × 10^−2^ at 0.5% *w*/*w* CMC. After subsequent incubation at pH 7.0 for 2 h, *Γ* declined further, reaching 0.99–6.33 mg·m^−2^ × 10^−2^ depending on the formulation. A comparable trend was observed for LDE, but Γ values were consistently lower than in SDE across all CMC concentrations. For instance, at 0.1% *w*/*w* CMC, Γ decreased from 2.20 initially to 1.20 mg·m^−2^ × 10^−2^ at pH 2.0 and 1.02 mg·m^−2^ × 10^−2^ at pH 7.0, while at higher CMC levels (0.3–0.5% *w*/*w*) the corresponding reductions were from 5.6–6.6 to 3.9–5.0 mg·m^−2^ × 10^−2^ following transfer to intestinal conditions. Statistical analysis (one-way ANOVA with Tukey’s test) confirmed significant reductions in interfacial CMC concentration (*Γ*) after exposure to both gastric and intestinal conditions (*p* < 0.05), particularly between initial and post-gastric/post-intestinal stages. Differences between gastric and intestinal stages were insignificant (Figure 3E,F).

These results indicate that CMC desorption from the droplet interface occurs progressively during sequential exposure to gastric and intestinal pH conditions, reflecting the polymer’s pH sensitivity. In acidic environments, partial protonation of carboxyl groups reduces the solubility of carboxymethyl cellulose (CMC), weakening its electrostatic affinity for the oil–water interface [8]. The subsequent transition to neutral pH increases CMC ionisation, promoting further desorption due to enhanced electrostatic repulsion [42].

Despite these changes, *Γ* remained dependent on the initial polymer concentration, suggesting that higher CMC levels favour the formation of denser interfacial structures that are more resistant to desorption. The overall *Γ* pattern followed the initial > pH 2.0 > pH 7.0 order for both emulsion types. Although SDE exhibited higher absolute *Γ* values than LDE, the relative change (|%Δ*Γ*|) did not differ significantly between emulsion types (two-way ANOVA, *p* > 0.05; Figure S6), suggesting comparable CMC desorption behaviour during pH transition.

The interfacial adsorption of CMC provides a mechanistic basis for the stabilising effects described earlier. Even though the ζ-potential decreased under acidic conditions, the presence of an adsorbed CMC layer maintained interfacial separation through combined steric and electrostatic repulsion. This electrosteric barrier effectively prevented droplet aggregation and coalescence during exposure to variable pH. In contrast, emulsions lacking CMC, where surface charge approached neutrality, exhibited marked instability, confirming the essential role of interfacial polymer adsorption in preserving emulsion integrity throughout gastrointestinal pH transitions.

#### 3.2.4. pH-Controlled Release of Encapsulated Compounds Under Simplified Gastric and Intestinal Physiological and Pathological Conditions

**Figure 4:**
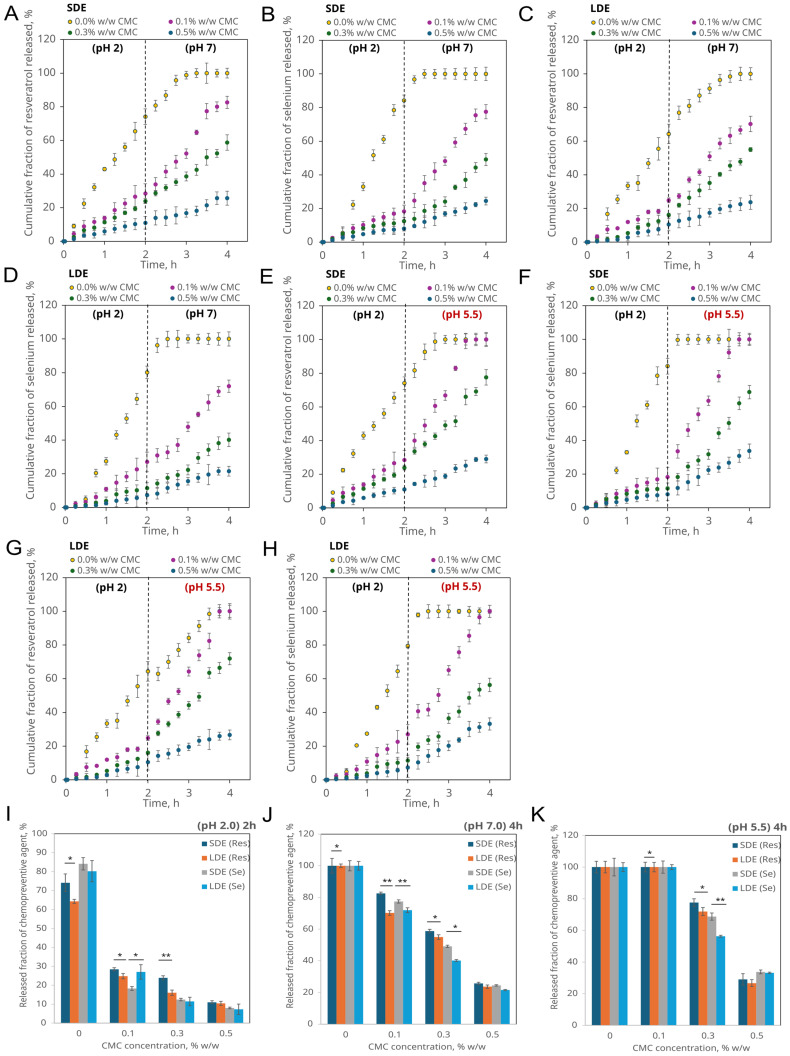
pH-controlled release of resveratrol (Res) and selenium (Se) from CMC-stabilised multiple emulsions during simulated gastrointestinal pH transitions. Panels (**A**–**D**) show the release profiles for the pH transition 2.0 → 7.0, corresponding to the simulated passage from the gastric to the physiological intestinal environment: (**A**) resveratrol release from SDE, (**B**) selenium release from SDE, (**C**) resveratrol release from LDE, (**D**) selenium release from LDE. Panels (**E**–**H**) represent the pH transition 2.0 → 5.5, simulating pathological intestinal conditions: (**E**) resveratrol release from SDE, (**F**) selenium release from SDE, (**G**) resveratrol release from LDE, (**H**) selenium release from LDE. Panels (**I**–**K**) present the released fractions of resveratrol and selenium after sequential exposure to simulated gastrointestinal conditions: 2 h at pH 2.0 (gastric stage) followed by a pH transition to either 7.0 (physiological) or 5.5 (pathological intestinal environment) for an additional 2 h. Each value represents the mean ± SD (error bars not visible, mean errors are the same size, or smaller, than the symbol for a given measurement). Statistically significant differences between SDE and LDE are indicated by *p* < 0.05 (*) and *p* < 0.01 (**) (one-way ANOVA with Tukey’s test).

The CMC-stabilised multiple emulsions, containing sodium selenite in the internal aqueous phase droplets and trans-resveratrol in the oil membrane drops, exhibited a two-step, pH-responsive pattern corresponding to the simulated gastric (0–2 h, pH 2.0) and intestinal (2–4 h, pH 7.0 or 5.5) phases (Figure 4A–H).

At pH 2.0 (simulated gastric stage), the release of both compounds remained limited. In CMC-free emulsions, cumulative release after 2 h reached approximately 62–75% for resveratrol and 80–83% for selenium. Increasing CMC concentration led to a marked reduction in release, with values below 10–15% at 0.5% *w*/*w* CMC. This reflects the higher viscosity and compactness of the protonated CMC layer under acidic conditions, which restricts diffusion of encapsulated molecules from the internal droplets and through the interfacial film. LDE consistently showed slower release than SDE due to their larger droplet size and smaller specific interfacial area.

Following pH adjustment to 7.0, mimicking physiological intestinal conditions, the cumulative release increased across all formulations (Figure 4A–D). The release fraction remained inversely proportional to interfacial CMC concentration (see Section 3.2.3). After 4 h, SDE without CMC released nearly 100% of both compounds, whereas systems containing 0.3–0.5% CMC released only 22–58% (resveratrol) and 25–45% (selenium). The overall release from LDE followed the same trend but was significantly slower (*p* < 0.05–0.01, one-way ANOVA with Tukey’s test; Figure 4J).

The differences between the two emulsion types can be attributed to droplet geometry: smaller droplets provide a greater interfacial area and shorter diffusion distance, facilitating release once the CMC film becomes partially relaxed at neutral pH.

Under mildly acidic (pH 5.5) simplified pathological intestinal conditions, mimicking inflammation, the release of both compounds increased relative to pH 7.0 (Figure 4E–H). After 4 h, CMC-free emulsions reached complete (≈100%) or nearly complete release, while 0.5% CMC systems achieved 22–30%. This suggests that partial CMC ionisation at pH 5.5 weakens polymer–oil interactions, producing a more permeable interfacial film. The effect was more pronounced in SDE, whose thinner oil layers promote faster diffusion, whereas LDE maintained significantly lower release (*p* < 0.05; Figure 4K).

Across all conditions, increasing CMC concentration consistently limited release (ANOVA, *p* < 0.01). Mean release values (Figure 4I–K) confirmed that SDE > LDE under every CMC concentration, with the most significant differences at 0.1–0.3% CMC (*p* < 0.05–0.01). Selenium release was systematically lower than that of resveratrol, consistent with its localisation in the internal aqueous phase, while resveratrol resided in the oil membrane.

The results indicate that the release of both chemopreventive agents is primarily governed by droplet size, interfacial polymer density, and the pH-dependent structure of CMC in the external phase. These observations were obtained under enzyme- and bile salt-free conditions, representing a simplified model system to isolate pH-dependent phenomena. However, enzymatic activity and bile salt interactions are known to affect emulsion stability and release kinetics; therefore, future work will involve testing under biorelevant gastrointestinal media to capture these additional effects.

### 3.3. Mucoadhesive Properties of Chemopreventive Agents-Loaded Emulsions

**Figure 5:**
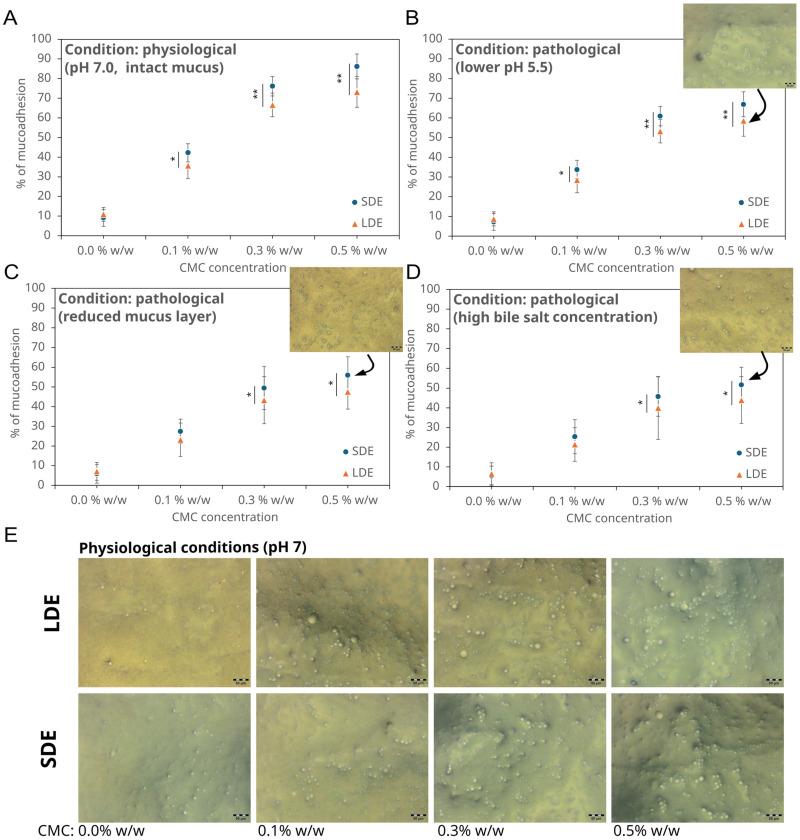
Mucoadhesive properties of small-droplet (SDE) and large-droplet (LDE) multiple emulsions: percentage of mucoadhesion of emulsion with different sodium carboxymethyl cellulose (CMC) concentrations under physiological (pH 7.0, intact mucus- (**A**)) and pathological conditions (pH 5.5 (**B**), reduced mucus layer (**C**), and high bile salt concentration (**D**)), (Each value represents the mean ± SD, error bars not visible, mean errors are the same size, or smaller, than the symbol for a given measurement), asterisks denote statistically significant differences between SDE and LDE at the same CMC concentration (*p* < 0.05 (*), *p* < 0.01 (**); one-way ANOVA with Tukey’s test); (**E**) representative micrographs of porcine intestinal epithelium showing adsorbed emulsion droplets, appearing as brighter areas on the mucosal surface, under physiological conditions (pH 7.0).

The mucoadhesion of emulsions containing the chemopreventive agents plays a critical role in determining their potential residence time in the intestinal tract and may, consequently, contribute to improved local retention and bioavailability. As shown in Figure 5A, both droplet size and sodium carboxymethyl cellulose (CMC) concentration influenced the mucoadhesion percentage. In the absence of CMC, both SDE and LDE exhibited low mucoadhesion values (<10%), reflecting weak interactions with the mucus layer. Increasing the CMC concentration from 0.1% *w*/*w* to 0.5% *w*/*w* markedly enhanced adhesion for both emulsion types, LDE and SDE.

The improvement was more pronounced in SDE, which reached adhesion values close to 90% at 0.5% *w*/*w* CMC, compared with approximately 75% for LDE at the same concentration. The stronger response of SDE may be attributed to the greater surface area provided by smaller droplets, which facilitates interactions between the polymer chains of CMC and mucin. Similar observations on the importance of particle surface area in promoting effective mucoadhesion have been reported previously [43,44]. At higher polymer concentrations, however, the increase in mucoadhesion became less pronounced, with only a modest rise observed between 0.3% *w*/*w* and 0.5% *w*/*w* CMC for both emulsions. This behaviour may reflect a saturation effect, in which the addition of polymer (CMC) does not substantially increase adhesion due to limited availability of binding sites or reduced accessibility of polymer chains within the mucus layer, consistent with findings reported in other mucoadhesive systems [34,45].

In addition to physiological intestinal conditions (pH 7.0, intact mucus), experiments were conducted under selected pathological simulations. At lower pH (5.5) (Figure 5B), intended to mimic inflammatory states of the small intestine, mucoadhesion decreased compared with the physiological condition, particularly at higher CMC levels. This may be explained by reduced ionisation of the carboxyl groups in CMC, which diminishes electrostatic interactions with mucin [32]. Similarly, the reduction in the mucus layer led to a pronounced decrease in adhesion, consistent with reports describing the thinning of intestinal mucus gels in inflammatory bowel disease [33]. Excess bile salts, simulating malabsorption syndromes or bile reflux, also markedly reduced mucoadhesion, most likely through the solubilisation of lipid droplets and destabilisation of the mucus structure [46].

The most significant deviations from physiological conditions were observed under reduced mucus layer (Figure 5C) and bile salt excess (Figure 5D) simulations, where adhesion decreased by approximately 35–40% compared with the intact state. In contrast, lowering the pH (5.5) or adding proteolytic enzymes produced more moderate reductions (15–25%). Despite these decreases, the percentage of mucoadhesion remained relatively high for systems containing 0.5% *w*/*w* CMC, particularly for SDE, which consistently achieved adhesion values above 50% across all tested pathological conditions. These results suggest that adequate polymer levels can partially offset adverse environmental factors, thereby maintaining meaningful interaction with the intestinal mucus.

Microscopic observations confirmed these quantitative results. Under ex vivo physiological conditions (pH 7.0, intact mucus) (Figure 5E), numerous emulsion droplets adhered to the epithelial surface, forming a continuous layer. Under mildly acidic and pathological conditions (pH 5.5, reduced mucus layer, or in the presence of excess bile salts), the number of attached droplets decreased, indicating partial loss of mucoadhesion. Nonetheless, systems containing higher CMC concentrations retained significant attachment, particularly the SDE formulations. These findings demonstrate that both the polymer content and droplet size govern the extent of adhesion, and that CMC effectively promotes mucoadhesive interactions even under compromised intestinal environments in the ex vivo model examined.

## 4. Conclusions

Sodium carboxymethyl cellulose (CMC) effectively stabilised W/O/W multiple emulsions designed as a model oral delivery system for trans-resveratrol and selenium intended for chemopreventive application in colorectal cancer. Increasing CMC concentration enhanced the physical stability of the emulsions by reducing droplet coalescence, increasing viscosity, and providing electrosteric protection. The systems exhibited pH-responsive behaviour, limiting release under acidic conditions, mimicking gastric fluid, while enabling controlled diffusion at neutral and mildly acidic intestinal pH. Higher CMC levels also improved mucoadhesion, particularly in small-droplet emulsions (SDE), maintaining significant adhesion even under simulated pathological environments.

The present study was conducted using simplified gastrointestinal models (enzyme- and bile salt-free) and an ex vivo intestinal adhesion system, allowing controlled evaluation of pH-dependent stability, compound release, and mucoadhesive behaviour. While these controlled conditions provided mechanistic insight into pH-dependent interfacial phenomena, further investigations using fully biorelevant gastrointestinal media, together with dynamic digestion models, are required, as these factors can alter emulsion integrity, chemical stability of the encapsulated compounds, release behaviour, and overall bioaccessibility, thereby determining the in vivo performance of the system.

The findings of this study indicate that CMC plays a pivotal role in maintaining the structural integrity, pH-responsive release, and mucoadhesive potential of multiple emulsions, confirming their suitability as a model platform for oral co-delivery of chemopreventive agents. Moreover, this work provides novel mechanistic insight into the adaptive interfacial behaviour of hydrocolloid-stabilised multiple emulsions under variable pH conditions, bridging formulation design with their potential functional performance in simulated gastrointestinal environments.

## Figures and Tables

**Table 1:** The composition of the multiple emulsion phases.

Phase	Composition
Internal	Gelatin (0.2% *w*/*w*), Pluronic P-123 (0.25% *w*/*w*), Milli-Q water (to 100% *w*/*w*)
Membrane	Sesame oil (98% *w*/*w*), Span 83 (2% *w*/*w*)
External	Sodium carboxymethyl cellulose—CMC (0.0–0.5% *w*/*w*), Pluronic P-123 (0.25% *w*/*w*), Tween 80 (0.25% *w*/*w*), Milli-Q water (to 100% *w*/*w*)

**Table 2:** Preparation conditions of the emulsions in a Couette–Taylor flow (CTF) contactor.

Emulsion Series	CMC Content % *w*/*w*	Rotational Frequency rpm	Flow Rate of the Emulsion Phases		
			Internal cm^3^ min^−1^	Membrane cm^3^ min^−1^	External cm^3^ min^−1^
SDE ^1^	0.0	900	30	30	60
	0.1	1200			
	0.3	1400			
	0.5	1800			
LDE ^2^	0.0	1300		60	
	0.1	1800			
	0.3	2100			
	0.5	2450			

^1^ SDE—small-droplet emulsion, ^2^ LDE—large droplet emulsion.

## Data Availability

Data and results were reported in this article and Supplementary Materials.

## Supplementary Materials

The following supporting information can be downloaded at: https://www.mdpi.com/article/10.3390/pharmaceutics17111401/s1, Figure S1. Calibration curves for trans-resveratrol at pH (A) 2.0, (B) 5.5 and (C) 7.0 (PBS, 305 nm). Linear regression equations and R^2^ values are given in Table S1. Data represent mean ± SD (*n* = 3), (*p* > 0.05). Points represent error bars; not visible, mean errors are the same size or smaller than the symbol for a given measurement.; Table S1: Calibration parameters for trans-resveratrol at different pH values.; Figure S2. Calibration curve for Se(IV)–MBT complex at 370 nm (mean ± SD, *n* = 3). Points represent error bars; not visible, mean errors are the same size or smaller than the symbol for a given measurement. Linear regression: *A* = 0.003 + 0.1574 *c* (µg·cm^−3^); R^2^ = 0.999, (*p* > 0.05).; Table S2. One-way ANOVA results confirming the linearity of the Se(IV)–MBT spectrophotometric calibration model; Table S3. Precision and accuracy of the MBT spectrophotometric method for Se(IV) determination.; Figure S3. Residuals of the calibration fit for Se(IV)–MBT complex showing random distribution around zero, confirming linearity.; Figure S4. Percentage change in droplet diameter (|%Δ*D*_32_| = (*D*_32__*intestinal* − *D*_32__*initial*)/*D*_32__*initial* × 100) for small-droplet (SDE) and large-droplet (LDE) emulsions containing various concentrations of CMC. Bars represent mean ± SD (*n* = 3). Asterisks indicate significant differences between SDE and LDE at the same CMC level (*p* < 0.05; two-way ANOVA with Tukey’s test).; Figure S5. Relative change in ζ-potential (|%Δζ|= (ζ_intestinal − ζ_initial)/ζ_initial × 100) for small-droplet (SDE) and large-droplet (LDE) emulsions containing different concentrations of CMC after transition from initial (pH 7.4) to intestinal conditions (pH 7.0). Bars represent mean ± SD (*n* = 3). Asterisks indicate significant differences between SDE and LDE at 0.0 % *w*/*w* CMC (*p* < 0.05 (*); two-way ANOVA with Tukey’s test).; Figure S6. Relative change in interfacial CMC (|%Δ*Γ*||= (*Γ_intestinal* − *Γ_initial*)/*Γ_initial* × 100) for SDE and LDE emulsions after exposure to simulated gastrointestinal pH transition (intestinal – initial). Values are presented as mean ± SD (*n* = 3). Statistical analysis (two-way ANOVA with Tukey’s test) showed no significant differences between emulsion types (*p* > 0.05).
