# Supplementary material for: Sodium Carboxymethyl Cellulose-Stabilised Multiple Emulsions with pH-Sensitive Behaviour, Enhanced Stability and Mucoadhesion for Oral Delivery of Chemopreventive Agents
Source: Pharmaceutics. 2025 Oct 29;17(11):1401. doi: 10.3390/pharmaceutics17111401 (PMC12654994; doi:10.3390/pharmaceutics17111401)
Supplement: Supplementary file 1 [file pharmaceutics-17-01401-s001.zip › pharmaceutics-3946051-supplementary.pdf]

Supplementary Materials

Sodium Carboxymethyl Cellulose-Stabilised Multiple Emulsions with pH-Sensitive Behaviour, Enhanced Stability and Mucoadhesion for Oral Delivery of Chemopreventive Agents

Agnieszka Markowska-Radomska [1,*], Konrad Kosicki [2] and Ewa Dluska [1]

[1] Faculty of Chemical and Process Engineering, Warsaw University of Technology, Warynskiego 1, 00-645 Warsaw, Poland; ewa.dluska@pw.edu.pl
[2] Institute of Genetics and Biotechnology, Faculty of Biology, Warsaw University, Miecznikowa 1, 02-096 Warsaw, Poland; km.kosicki@uw.edu.pl
* Correspondence: agnieszka.markowska@pw.edu.pl

S1. Calibration and validation of the spectrophotometric assay for trans-resveratrol

Trans-resveratrol calibration curves were established in phosphate-buffered saline (PBS) and adjusted to pH 2.0, 5.5, and 7.0. Fresh standard solutions (1–20 μg·cm^{-3}) were prepared in each buffer immediately before measurement and protected from light. Absorbance was measured at 305 nm against a pH-matched blank using 1 cm quartz cuvettes using a Jasco FP-6500 spectrophotometer (Japan). Calibration curves (A_{305} vs concentration) were constructed by least-squares linear regression (Figure S1). Limits of detection (LOD) and quantification (LOQ) were estimated as 3.3σ/S and 10σ/S, respectively (σ — the standard deviation, S — the slope of the calibration curve (from linear regression)), Table S1.

(A)

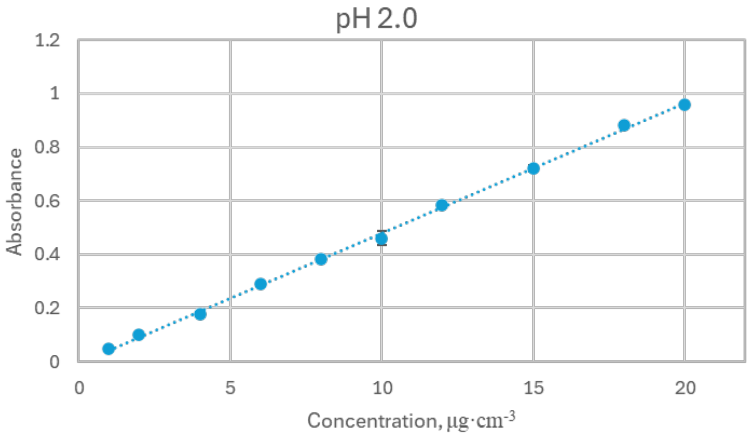

(B)

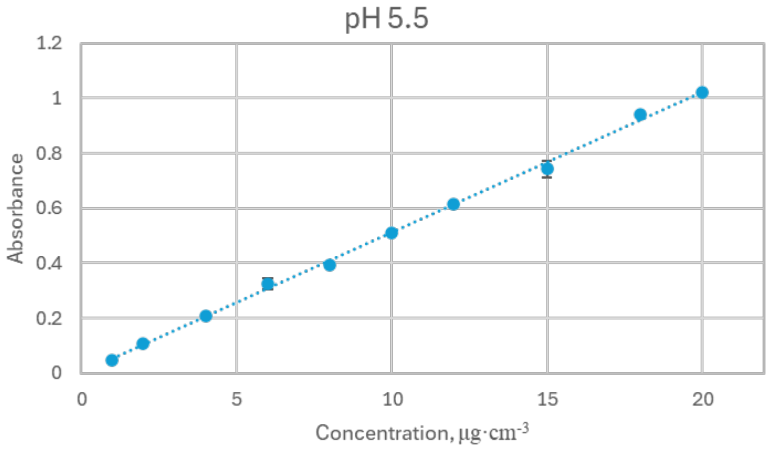

(C)

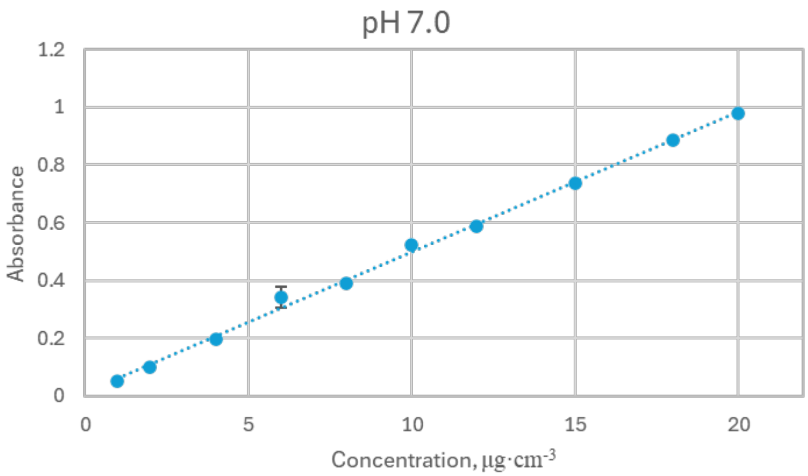

Figure S1. Calibration curves for trans-resveratrol at pH (A) 2.0, (B) 5.5 and (C) 7.0 (PBS, 305 nm). Linear regression equations and R^2 values are given in Table S1. Data represent mean ± SD (n = 3), ($p > 0.05$). Points represent error bars; not visible, mean errors are the same size or smaller than the symbol for a given measurement.

Table S1. Calibration parameters for trans-resveratrol at different pH values

pH	Linear range µg·cm^{-3}	Regression equation A = aC + b	R^2	LOD µg·cm^{-3}	LOQ µg·cm^{-3}
2.0	1-20	A = 0.048C + 0.002	0.996	0.20	0.60
5.5	1-20	A = 0.051C + 0.001	0.998	0.18	0.55
7.0	1-20	A = 0.049C + 0.002	0.997	0.19	0.57

A — absorbance of the solution, C — concentration of trans-resveratrol (µg·cm^{-3}).

Regression equations obtained from calibration curves were used to determine trans-resveratrol concentrations in the external phase of multiple emulsions under different pH conditions.

Statistical evaluation: One-way ANOVA (factor: trans-resveratrol concentration, 1–20 µg·cm^{-3}) confirmed a significant effect of concentration on absorbance at 305 nm for all tested pH values ($p < 0.0001$). No significant lack-of-fit was observed ($p > 0.05$), and residuals were randomly distributed around zero, confirming the linearity of the calibration models. Residuals for calibration curves (pH 2.0, 5.5, 7.0) showed no systematic deviation; therefore, the models were considered valid within the working range (1–20 µg·cm^{-3}).

S2. Calibration curve for selenium

Selenium, originating from sodium selenite, was determined using a modified spectrophotometric procedure of Bera and Chakrabartty (1968, Analyst, 93, 50–53). The method is based on forming a yellow Se(IV)–2-mercaptobenzothiazole (MBT) complex in a strongly acidic medium, measurable at 370 nm. The pH of the samples was first adjusted to 7.5 ± 0.2 with 0.1 M HCl/NaOH to promote complex formation. For the assay, 1 cm^3 of the sample or standard was mixed with 15 cm^3 of 12 M HCl and 4 cm^3 of 0.1 % (w/v) MBT solution in 50 % ethanol, then diluted with 2 M HCl to a final

volume of 20 cm^3. The reaction mixture was vortexed, incubated for 10 min at room temperature, and the absorbance (A_{370}) was measured against a reagent blank using a 1 cm quartz cuvette.

Calibration standards were prepared from sodium selenite in the range 0–8 μg·cm^{-3} Se(IV) in a matrix-matched blank (PBS mixed with the emulsion external phase at a 10:1 (v/v) ratio, adjusted to pH 7.5, and treated identically to the samples). The calibration curve followed the equation:

$$A = (0.003 \pm 0.004) + (0.1574 \pm 0.0008) \cdot c$$

with $R^2 = 0.9989$ for eight points (0.5–8 μg·cm^{-3}), ($p > 0.05$; no deviation from linearity) (Figure S2).

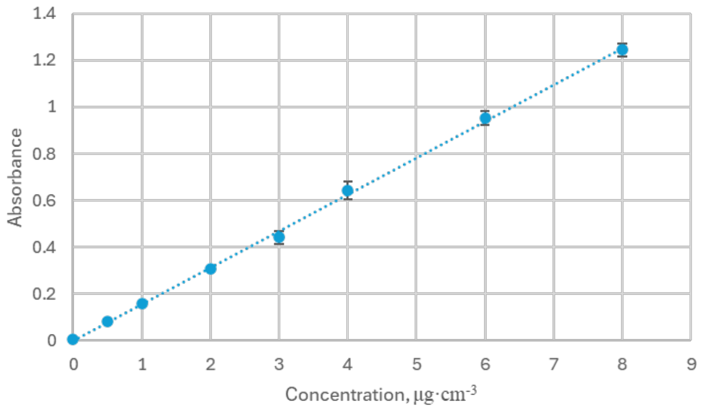

Figure S2 Calibration curve for Se(IV)–MBT complex at 370 nm (mean ± SD, n = 3). Points represent error bars; not visible, mean errors are the same size or smaller than the symbol for a given measurement. Linear regression: A = 0.003 + 0.1574 c (μg·cm^{-3}); $R^2 = 0.999$, ($p > 0.05$).

The calibration range covered the expected selenium concentrations released from emulsions under all tested conditions."

One-way ANOVA (factor: Se(IV) concentration) was applied to verify the significant effect of concentration on absorbance as an additional confirmation, Table S2.

Table S2. One-way ANOVA results confirming the linearity of the Se(IV)–MBT spectrophotometric calibration model.

Source	df	SS	MS	F	p-value
Between concentrations	7	2.252	0.322	974.3	< 0.0001
Within (error)	16	0.0053	0.00033		
Total	23	2.257			

df – degrees of freedom; SS – sum of squares; MS – mean square; F – F-statistic

The analysis confirmed a statistically significant effect of Se(IV) concentration on absorbance ($F(7,16) = 974.3$, $p < 0.0001$). No lack-of-fit was detected ($p > 0.05$), confirming a linear response within the working range.

The limit of detection (LOD) and limit of quantification (LOQ) were calculated as "LOD"=3.3·σ/b, "LOQ"=10·σ/b, where σ = standard deviation of 10 blank readings (0.0043 AU) and b = slope of the calibration curve, yielding LOD = 0.076 μg·cm^{-3} and LOQ = 0.23 μg·cm^{-3}.

Precision and accuracy results are summarised in Table S3.

Table S3. Precision and accuracy of the MBT spectrophotometric method for Se(IV) determination.

Target spike μg·cm^{-3}	Found (mean ± SD) μg·cm^{-3}	Recovery (%)	RSD (%)
0.5	0.49 ± 0.02	98	4.1
2.0	2.02 ± 0.05	101	2.5
6.0	5.88 ± 0.18	98	3.1

Repeatability (3 μg·cm^{-3}, n = 6): mean = 3.02 μg·cm^{-3}, RSD = 0.9 %. All acceptance criteria were met ($R^2 \geq 0.995$; RSD ≤ 5 %; recovery 95–105 %; LOD ≤ 0.1 μg·cm^{-3}).

Robustness and interferences: Small variations in reaction time (8–12 min) or temperature (20–25°C) caused a ≤ 3 % change in absorbance. Matrix blanks were used for background correction. No drift in A_{370} was observed within 60 min. Sample pH (7.5 ± 0.2) was adjusted prior to acidification; no carry-over was observed.

Residual analysis confirmed the linearity of the calibration model (Figure S3).

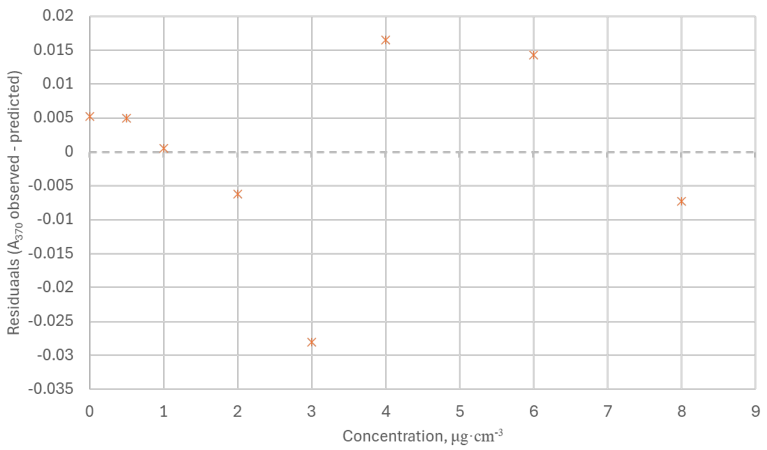

Figure S3 Residuals of the calibration fit for Se(IV)–MBT complex showing random distribution around zero, confirming linearity.

S3. Relative change in droplet size (|%ΔD_{32}|) during simulated gastrointestinal pH transition

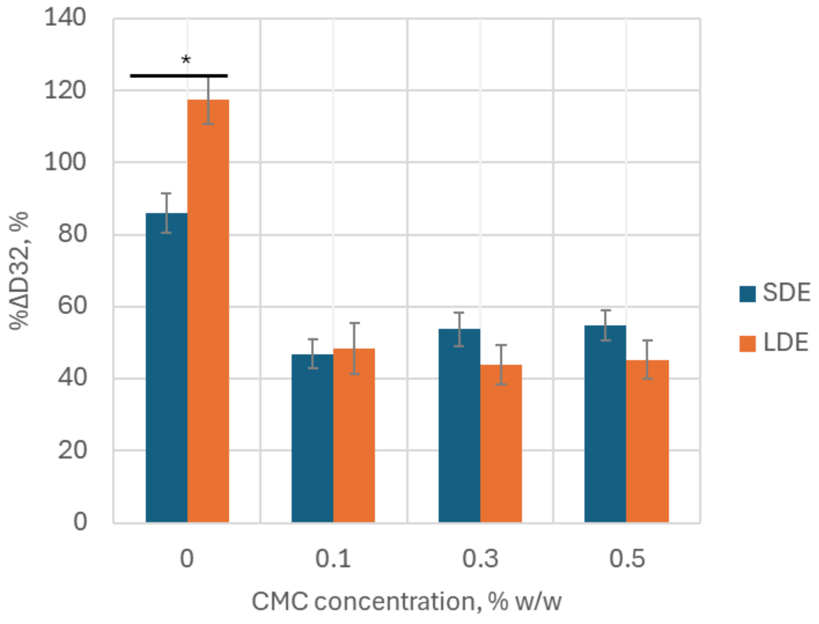

Figure S4 Percentage change in droplet diameter ($|\%\Delta D_{32}|$ = (D_{32}_intestinal − D_{32}_initial)/D_{32}_initial × 100) for small-droplet (SDE) and large-droplet (LDE) emulsions containing various concentrations of CMC. Bars represent mean ± SD (n = 3). Asterisks indicate significant differences between SDE and LDE at the same CMC level ($p < 0.05$; two-way ANOVA with Tukey's test).

S4. Relative change in ζ-potential during simulated gastrointestinal pH transition

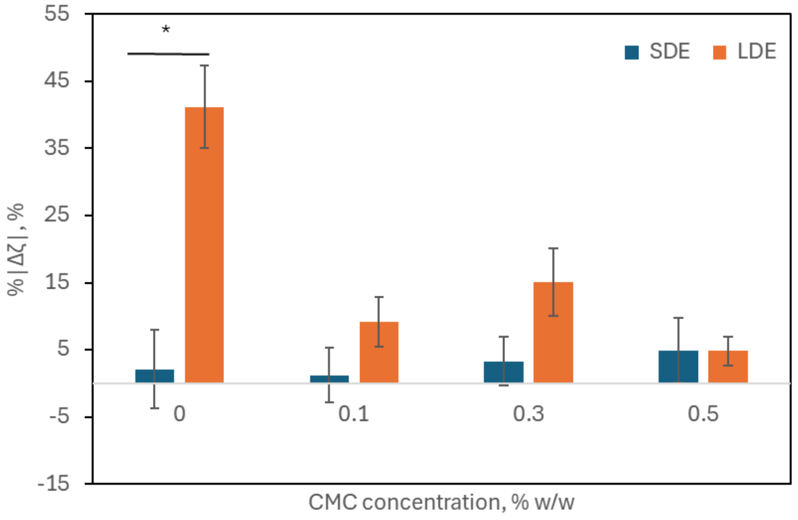

Figure S5. Relative change in ζ-potential ($|\%\Delta\zeta|$= (ζ_intestinal − ζ_initial)/ ζ_initial × 100) for small-droplet (SDE) and large-droplet (LDE) emulsions containing different concentrations

of CMC after transition from initial (pH 7.4) to intestinal conditions (pH 7.0). Bars represent mean ± SD (n = 3). Asterisks indicate significant differences between SDE and LDE at 0.0 % w/w CMC (* $p < 0.05$; two-way ANOVA with Tukey's test).

S5. Relative change in interfacial CMC (|%ΔΓ|) during simulated gastrointestinal pH transition

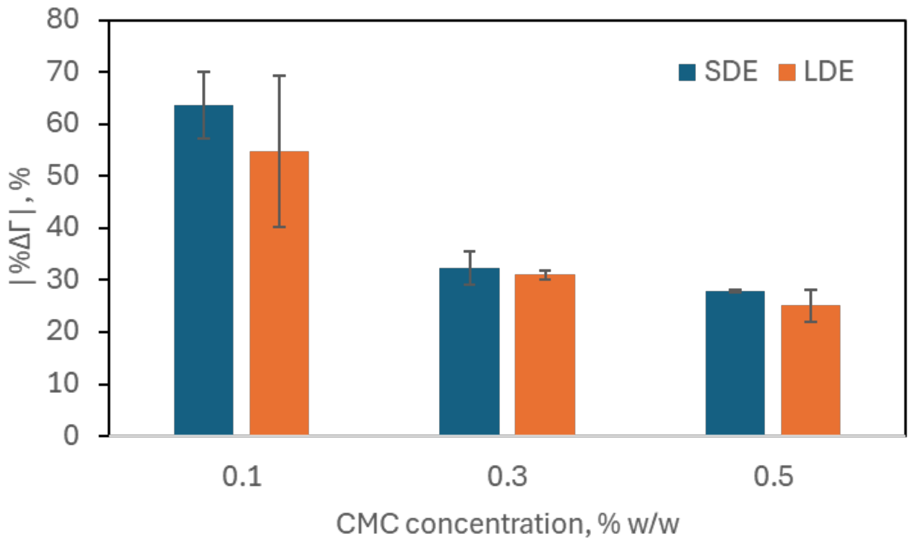

Figure S6. Relative change in interfacial CMC (|%ΔΓ||= (Γ_intestinal − Γ_initial)/ Γ_initial × 100) for SDE and LDE emulsions after exposure to simulated gastrointestinal pH transition (intestinal − initial). Values are presented as mean ± SD (n = 3). Statistical analysis (two-way ANOVA with Tukey's test) showed no significant differences between emulsion types ($p > 0.05$).
